# Using Population Mobility Patterns to Adapt COVID-19 Response Strategies in 3 East Africa Countries

**DOI:** 10.3201/eid2813.220848

**Published:** 2022-12

**Authors:** Rebecca D. Merrill, Fadhili Kilamile, Mwabi White, Daniel Eurien, Kanan Mehta, Joseph Ojwang, Marianne Laurent-Comlan, Peter Ahabwe Babigumira, Lydia Nakiire, Alexandra Boos, Wangeci Gatei, Julie R. Harris, Alain Magazani, Felix Ocom, Robert Ssekubugu, Godfrey Kigozi, Florent Senyana, Francis B. Iyese, Peter James Elyanu, Sarah Ward, Issa Makumbi, Allan Muruta, Elvira McIntyre, Khalid Massa, Alex R. Ario, Harriet Mayinja, Kakulu Remidius, Dede N. Ndungi

**Affiliations:** US Centers for Disease Control and Prevention, Atlanta, Georgia, USA (R.D. Merrill, K. Mehta, J. Ojwang, W. Gatei, J.R. Harris, S. Ward);; Tanzania Ministry of Health, Dodoma, Tanzania (F. Kilamile, K. Massa, K. Remidius);; Democratic Republic of the Congo Ministry of Health, Kinshasa, Democratic Republic of the Congo (M. White, F.B. Iyese, D.N. Ndungi);; Baylor College of Medicine Children’s Foundation Uganda, Kampala, Uganda (D. Eurien, P.J. Elyanu);; Bizzell Group, Kinshasa (M. Laurent-Comlan, F. Senyana); Infectious Diseases Institute, Kampala (P.A. Babigumira, L. Nakiire);; Agency for Toxic Substances and Disease Registry, Atlanta, Georgia, USA (A. Boos, E. McIntyre);; US Centers for Disease Control and Prevention, Dar es Salaam, Tanzania (W. Gatei);; African Field Epidemiology Network, Kinshasa (A. Magazani);; Uganda Ministry of Health, Kampala (F. Ocum, I. Makumbi, A. Muruta, H. Mayinja);; Rakai Health Sciences Program, Kalisizo, Uganda (R. Ssekubugu, G. Kigozi);; National Public Health Institute, Kampala (A.R. Ario)

**Keywords:** COVID-19, SARS-CoV-2, coronavirus disease, severe acute respiratory syndrome coronavirus 2, respiratory infections, zoonoses, viruses, international health, border crossing, population dynamics, community networks, PopCAB, Democratic Republic of the Congo, DRC, Tanzania, Uganda

## Abstract

The COVID-19 pandemic spread between neighboring countries through land, water, and air travel. Since May 2020, ministries of health for the Democratic Republic of the Congo, Tanzania, and Uganda have sought to clarify population movement patterns to improve their disease surveillance and pandemic response efforts. Ministry of Health–led teams completed focus group discussions with participatory mapping using country-adapted Population Connectivity Across Borders toolkits. They analyzed the qualitative and spatial data to prioritize locations for enhanced COVID-19 surveillance, community outreach, and cross-border collaboration. Each country employed varying toolkit strategies, but all countries applied the results to adapt their national and binational communicable disease response strategies during the pandemic, although the Democratic Republic of the Congo used only the raw data rather than generating datasets and digitized products. This 3-country comparison highlights how governments create preparedness and response strategies adapted to their unique sociocultural and cross-border dynamics to strengthen global health security.

Border health systems are structured to prevent, detect, and respond to and mitigate the effects of public health events among mobile populations, notably those traveling across international boundaries (*1*). Throughout the COVID-19 pandemic, cross-border air travel and movement over land and water helped drive the international spread of SARS-CoV-2. National and local government agencies, global public health partners, and private sector stakeholders implemented various border health mitigation measures, which included screening at international point of entry (POE) locations and at domestic point of control (POC) locations in communities and priority locations along travel routes to limit the spread of COVID-19 (*2*).

Although data such as volume and destination are available for formally documented travel by plane or cruise ship, informal traveler movement (i.e., by private conveyance or across porous borders) provides less data for analysis and decision making. Scientists and public health practitioners continue to advance the use of social media and mobile phone data to understand mobility (*3*). The products from these analyses are very informative, but the capacity to create them is often not available in the areas affected by the movement. This deficiency of data or access to advanced analytic methods on international mobility limits the capacity of public health authorities to build strategies adaptable to the unique risks of disease translocation within and between countries.

The US Centers for Disease Control and Prevention (CDC) developed the Population Connectivity Across Borders (PopCAB) toolkit as a resource for governments and other stakeholders to gather and analyze data about population mobility to inform public health interventions (*4*). In brief, the toolkit provides template guides for focus group discussions (FGDs) and key informant interviews (KIIs), considerations for developing the base maps for participatory mapping, template materials, and techniques for managing and analyzing the data, and training materials on methods for preparing for, implementing, and applying the data from PopCAB activities. CDC can provide the toolkit to countries and partners along with technical assistance, as interested, throughout the process.

The Democratic Republic of the Congo (DRC), Tanzania, and Uganda ministry of health (MOH) offices sought to develop COVID-19 border health surveillance and mitigation measures better adapted to their unique community connectivity and population mobility patterns (*5*,*6*). To address its goal, each MOH, in collaboration with partners, implemented the PopCAB toolkit after adapting the template materials for FGDs and KIIs with participatory mapping to their country context (*7*). These community engagement activities and their associated analyses provided the implementers with a better understanding of population movement patterns. The countries applied the information to improve COVID-19 surveillance, testing, and border health policies. These COVID-19 response-focused PopCAB activities built on previous PopCAB efforts implemented in all 3 countries during the 2018–2020 Ebola virus disease epidemic in eastern DRC (*8*,*9*). We identified lessons learned and best practices by comparing the PopCAB initiatives the 3 MOHs implemented during the COVID-19 pandemic and how they applied the results.

## Methods

During May 2020–March 2022, MOH-led teams in DRC, Tanzania, and Uganda used the PopCAB toolkit to inform COVID-19 response strategies. As part of PopCAB, these teams, in collaboration with CDC and implementing partners in DRC and Uganda, conducted FGDs and KIIs with spatially-accurate participatory mapping to gather information about community-level, domestic, or cross-border population movement and connectivity patterns. 

To implement PopCAB, teams completed actions across 4 phases: preparation, characterization, visualization, and application. During the preparation phase, the team identified objectives and priority geographic areas or population groups of focus, adapted the PopCAB materials to address the objectives and context, worked with community leadership structures to identify stakeholders to invite to participate in FGDs and KIIs, and defined the timeline of activities. During the characterization phase, the team implemented as many PopCAB FGDs or KIIs as were needed to gather, process, and consolidate qualitative and spatial data. Depending on project objectives, the team could plan sessions around a specific event, such as a religious festival, or an important temporal rhythm, such as weekly during harvest season. To create the spatial dataset from the annotated maps and locations mentioned in the FGDs and KIIs, the teams geocoded each location of interest (LOI). LOIs represented origins, destinations, or locations along domestic and international travel routes, such as markets, health facilities, border towns, or transit towns. In the visualization phase, the team used the data from the characterization phase to identify, analyze, and visualize population movement and connectivity patterns by creating maps and narrative reports that illustrated population movement with respect to the LOI. Finally, the team used the application phase to adapt and improve programs and strategies with the results. Teams repeated these phases to address evolving public health needs, ensuring that they regularly identified opportunities to share experiences and develop plans to improve adapted materials and implementation approaches.

### Analyses

We compared the approaches that teams used to design and implement PopCAB. We compared, by country and PopCAB phase, details about the implementation timelines, team compositions, project goals, priority geographic areas or population groups of interest, data collection strategies, analytic approaches, and the application of results. To consolidate information for the comparative analysis, we gathered qualitative, quantitative, and spatial data from project implementation materials and outputs and qualitative data from discussions with the teams. Here, we intend to present a broad overview of PopCAB results from each country, rather than specific details.

## Results

**Figure 1:**
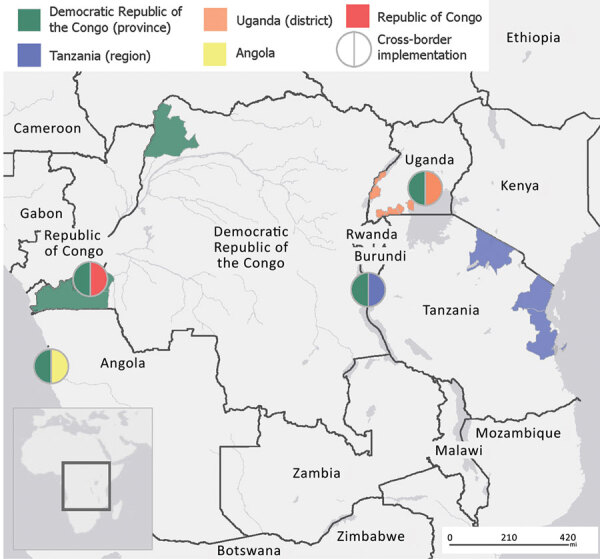
Areas where the Democratic Republic of the Congo, Tanzania, and Uganda ministries of health and their partners implemented Population Connectivity Across Borders events as part of COVID-19 control efforts, May 2020–March 2022.

During May 2020–March 2022, teams implemented 94 PopCAB events to inform COVID-19 response measures at binational, national, and local government and POE levels (Table 1; Figure 1): 8 in DRC; 24 in Tanzania; 60 in Uganda; 1 binational between DRC and Tanzania in Kigoma, Tanzania; and 1 binational between DRC and Uganda in Kampala, Uganda. Two of the 8 PopCAB events DRC implemented were also binational and conducted during cross-border meetings with Angola (in Luanda, Angola) and the Republic of Congo (in Kinshasa, DRC). Overall, the PopCAB participants in the 3 countries identified >2,000 unique LOIs through those discussions or associated annotations on the base maps.

**Table 1:** Characteristics of FGDs and KIIs implemented in 3 countries for COVID-19 response, May 2020–March 2022*

Country	No. FGD	No. KII	Participants’ job responsibilities or expertise	Geographic scale
DRC	6	0	Each FGD event included representation from the various services operating at the points of entry (POE): General Directorate of Migration, General Directorate of Customs and Excise, Border Police, National Border Hygiene Program (PNHF), National Intelligence Agency, naval force, lake police station, traders, truck drivers	All FGDs were implemented at POE; discussions focused on population movement through and around the POE
Cross-border DRC and Angola	1	0	Minister of Health of DRC, the International Health Regulations National Focal Point of each country, Director of epidemiologic surveillance in Angola, and the agents of the services operating at the borders of the two countries.	National-level FGD; discussion focused on cross-border population movement along the entire shared border
Cross-border DRC and Republic of Congo	1	0	The International Health Regulations National Focal Point of each country and the agents of the services operating at the border of the two countries	National-level FGD; discussion focused on cross-border population movement along the entire shared border with a focus on movement between the capitals of both countries
Tanzania	24	0	Each FGD event included an occupational group: Boda boda (motorcycle or bicycle) drivers (4), business persons (2), business women specifically (1), community leaders (2), dhow (wooden boat) operators (1), fishermen (2), healthcare providers (2), pastoralists (4), pastoralists and cattle traders (1), peasants (1), petty traders (1), salt producers (1), security officers (1), tour guides (1)	All FGDs were implemented at the subdistrict or ward level; Discussions focused on population movement into, through, and out of the administrative level 2 unit where the PopCAB event was being held
Uganda	34		Each event included an occupational group of representatives: Boda boda drivers (1), businesspersons (2), community outreach workers (1), community leaders (3), community persons (5), cultural leaders (2), customs officials (2), district leaders (2), health care workers (2), POE volunteers (2), security officers (2), sex workers (1), traders (2), traditional healers (2), transporters (2), truck drivers (2), village health volunteers (1)	FGDs and KIIs were implemented at district, village, or POE levels; discussions focused on population movement into, through, out of, and around the administrative area or local jurisdiction the person(s) represented
		26	Key informants were (persons with the same title represented different jurisdictions) border internal security officer for different border points (3), district internal security officer (2), deputy district internal security officer (1), district health educator (1), district health officer (1), District Police Commander (3), district surveillance focal person (1), herbalist (1), in-charge of immigration (1), liaison officer (1), local council (2), local council of defense (1), division commander (1), resident district commissioner (4), POE team lead for volunteer health screening (1), traditional healer (1)	
Cross-border DRC and Tanzania	1	0	International Health Regulations national focal points of the 2 countries, MOH representatives for border health and surveillance at national and regional or provincial levels of the 2 countries	National-level FGD; discussion focused on cross-border population movement along the entire shared border
Cross-border DRC and Uganda	1	0	Port Health or border health director of each country, IHR national focal point of DRC, representative of the minister of health for DRC, MOH representatives for border health and surveillance at national and district levels of the 2 countries, director and deputy director of Uganda’s National Public Health Institute, public health partners	National-level FGD; discussion focused on cross-border population movement along the entire shared border

*DRC, Democratic Republic of the Congo; FGD, focus group discussion; KII, key informant interview; POE, point of entry; PopCAB, Population Connectivity Across Borders.

### Preparation Phase

The national MOH port health director (in Tanzania and DRC) or border health focal point (in Uganda) led the PopCAB teams; their overall goal was to gather information about cross-border movement and community connectivity to inform border health strategies (Tables 2,3,4). Each team also included an International Health Regulations (2005) (*1*) national focal point; MOH national-level port health staff; district-level port health staff where available; government staff with epidemiology, public health surveillance, or emergency operations expertise; and Field Epidemiology Training Program or Field Epidemiology Laboratory Training Program residents. Government leadership for Uganda included the director and deputy director of the National Institute of Public Health. In Tanzania, MOH staff in collaboration with CDC conducted all activities. In DRC and Uganda, the governments led portions of activities independently; the remaining activities were conducted in collaboration with CDC and Africa Field Epidemiology Network; in Uganda, Baylor Uganda, Infectious Diseases Institute, and Rakai Health Sciences Program also supported activities.

**Table 2:** Components of the Population Connectivity Across Borders preparation phase decisions in 3 countries to inform COVID-19 response measures, May 2020–March 2022*

Component	DRC	Tanzania	Uganda
Implementation lead	MOH, PNHF, International Health Regulations national focal point	MOH Port Health program, International Health Regulations national focal point	MOH Border Health Program, International Health Regulations national focal point, and National Institute of Public Health
Partnerships	CDC, AFENET	CDC	CDC, Baylor Uganda, IDI, Rakai Health Sciences
Team members	National and provincial PNHF staff; FETP residents	MOH national, regional- and district-level officials from Port Health, the Emergency Operations Center, and Surveillance; FETP advisors, and residents	MOH Staff, FELTP mentors, and residents, District-level leadership
Objectives	Identify POE and POC (domestic) for enhanced and adjusted surveillance strategies to limit the spread of COVID-19 across international borders and provincial boundaries	Identify specific places of interest with population movement and connectivity patterns that may increase the risk for COVID-19 spread and other diseases	Tailor border health surveillance strategies for point of entry, informal crossing points, and cross-border communities
	Identify specific places and routes of interest with population movement and connectivity patterns that may influence the risk for spread of COVID-19 and other diseases through targeted interventions to enhance public health benefit and judicious use of resources	Tailor interventions to enhance public health benefit and judicious use of resource	Modify risk communication strategies for border communities
	Prioritize locations for enhanced staff training and surveillance	Prioritize POE, health facilities, and communities for enhanced staff training and surveillance	Prioritize POE, health facilities, and other locations for enhanced staff training and surveillance
	Identify secondary travel routes, including in formal border crossing points		Understand the influence of COVID-19 lockdowns on cross-border movement
	Identify sociodemographic characteristics of and means of travel among cross-border populations		Identify at-risk areas and populations
Priority geographic areas	Kinshasa, border regions, cross-border environments	Three regions along with Uganda and Kenya border	Western border with DRC, Southern border with Tanzania
Priority population groups	Persons moving across international and domestic administrative borders	Persons moving across borders with an emphasis on pastoralists and movement for animal herding	Mobile populations in general
First implemented for COVID-19 response	December 20	July 2020	May 2020

*AFENET, African Field Epidemiology Network; CDC, US Centers for Disease Control and Prevention; DRC, Democratic Republic of the Congo; FELTP, Field Epidemiology and Laboratory Training Program; FETP, Field Epidemiology Training Program; IDI, Infectious Diseases Institute; PNHF, Programme National d’Hygiène aux frontiers (National Border Health Program); POC, point of control; POE, point of entry; PopCAB, Population Connectivity Across Borders.

**Table 3:** Components of the Population Connectivity Across Borders preparation phase decisions in 2 cross-border national pairs to inform COVID-19 response measures, May 2020–March 2022*

Component	Binational: DRC and Uganda	Binational: DRC and Tanzania
Implementation lead	DRC PNHF, Uganda MOH	DRC PNHF, Tanzania MOH Port Health
Partnerships	US CDC, AFENET, Baylor Uganda, IDI, Uganda Public Health Fellowship Program	CDC, AFENET
Team members	National and district health and public health leadership	National and district health and public health leadership
Objectives	Identify similarities and differences in prioritized POE and border regions along shared border	Identify similarities and differences in prioritized points of entry and border regions along shared border
	Characterize cross-border movement to inform cross-border collaboration strategies	Characterize cross-border movement to inform cross-border collaboration strategies
Priority geographic areas	Shared DRC and Uganda border	Shared DRC and Tanzania border
Priority population groups	Cross-border mobile populations	Cross-border mobile populations
First implemented for COVID-19 response	September 2021	March 2022

*AFENET, African Field Epidemiology Network; CDC, US Centers for Disease Control and Prevention; DRC, Democratic Republic of the Congo; FELTP, Field Epidemiology and Laboratory Training Program; FETP, Field Epidemiology Training Program; IDI, Infectious Diseases Institute; PNHF, Programme National d’Hygiène aux frontiers (National Border Health Program); POC, point of control; POE, point of entry; PopCAB, Population Connectivity Across Borders.

**Table 4:** Comparison of 3 countries’ preparation phase decisions for conducting PopCAB to inform COVID-19 response measures, May 2020–March 2022*

Component	Similarities	Differences
Implementation lead	National MOH, Port Health, epidemiology	None
Partnerships	CDC	The Uganda team included many partners, whereas the other countries had teams predominantly or solely composed of MOH staff.
Team members	All countries invited national and district level MOH staff with a variety of expertise, e.g., surveillance, and emergency operations, to support implementation	None
Objectives	All teams implemented PopCAB to strengthen public health and border health systems and resource allocation	While DRC kept the objectives broader, with an interest in informing border health strategies, Uganda and Tanzania included more specific objectives, e.g., inform lockdown measures (Uganda) or explore cross-border animal movement (Tanzania)
Priority geographic areas	Border regions and urban areas visited by cross-border travelers	DRC focused specifically on POE and urban areas, while Tanzania and Uganda focused on administrative jurisdictions, e.g., county, district.
Priority population groups	Cross-border mobile populations	Uganda focused some activities on populations seeking traditional and formal healthcare support across a border. Tanzania focused some activities on populations that live mobile lives, e.g., pastoralists.
First implemented for COVID-19 response	All countries started implementing PopCAB for COVID-19 in 2020	While DRC focused on integrating PopCAB events throughout the pandemic, Uganda and Tanzania implemented intensive PopCAB initiatives at specific times and in specific areas

*CDC, US Centers for Disease Control and Prevention; MOH, ministry of health; PopCAB, Population Connectivity Across Borders.

**Figure 2:**
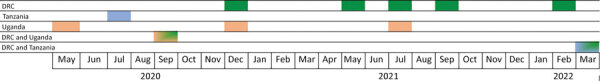
Timeline of Population Connectivity Across Borders implementation across Democratic Republic of the Congo (DRC), Tanzania, and Uganda during the COVID-19 response, May 2020–March 2022.

The teams implemented their PopCAB activities over 2 years of the COVID-19 response (Figure 2). DRC leveraged previously scheduled staff training, site visits, and cross-border meetings to implement events. In contrast, Uganda and Tanzania implemented PopCAB-focused initiatives to conduct many FGDs and KIIs in a short time. For example, Uganda implemented 30 events in Ntungamo, Isingiro, and Masaka districts near the Tanzania border in May 2020 after a disproportionate increase in COVID-19 cases in the region. Tanzania implemented 24 events in Arusha, Pwani, and Tanga Provinces in July 2020, recognizing the need to adapt COVID-19 response measures for continued cross-border movement and travel. Throughout the pandemic, the DRC border health director incorporated PopCAB events, or orientation to PopCAB, into all cross-border meetings the program joined or hosted. The DRC, Tanzania, and Uganda MOHs shared this accomplishment during bilateral meetings in Uganda with DRC in September 2021 and in Tanzania with DRC in March 2022 (Figures 1, 2).

Across these events, the teams in all 3 countries implemented PopCAB to address a few consistent objectives. One focused on a priority to adapt strategies for surveillance and risk communication at POEs and POCs to limit the spread of COVID-19 across international and domestic administrative boundaries. A second aimed to enhance public health benefit and judicious use of resources for surveillance and community outreach by identifying and prioritizing specific geographic areas visited by cross-border travelers. A third consistent objective was to prioritize secondary travel routes and areas of interest for mobile populations, including formal and informal POEs, health facilities, and community areas, for enhanced staff training and surveillance. The teams also implemented PopCAB to inform cross-border collaboration. Unique objectives included understanding the influence of COVID-19 lockdown measures on cross-border population movement in Uganda and addressing human activity associated with livestock husbandry in Tanzania.

### Characterization Phase

All teams implemented PopCAB events at national and community levels in administrative areas along international borders and in their countries’ large urban areas, e.g., Kinshasa, Dar es Salaam, and Kasese. DRC and Uganda also implemented events specifically at POEs; DRC implemented all their community-level events at POEs.

The teams invited various stakeholders to participate in PopCAB events, including security officers, truck drivers, traditional healers, village health volunteers, sex workers, and pastoralists (Table 1). These groups represented not only communities that may move across borders but also those that interact with travelers. Tanzania and Uganda implemented events with persons representing a variety of occupations and responsibilities relevant to a broader geographic scale, such as a district or region. Only Uganda implemented KIIs to gather more information from leadership or to address challenges with gathering multiple representatives for a group of interest, such as traditional healers, security personnel, and medical staff.

### Visualization Phase

The countries’ teams developed narratives and reports that listed all the LOIs and routes. The reports also described themes from the informal qualitative analyses completed by those who directly conducted the discussions or recorded notes. For example, these reports provided details about cross-border travel patterns to seek care from traditional healers, travel routes community members took when seeking healthcare to conceal residence status, or routes to avoid lockdown policies. The teams included photos of the annotated base maps and photos of participants during the events.

Each team followed a different approach to managing and analyzing the gathered information. The DRC team completed detailed reports rapidly, within 1 week, after each PopCAB event; however, they did not develop qualitative or spatial datasets of LOIs or routes for future visualizations and analyses. They took this approach because they intended to focus on applying results immediately after the discussions and their staff availability to process and manage the data over time was limited. 

The Tanzania team developed detailed FGD transcripts after each event to facilitate their ability to integrate and analyze results across multiple PopCAB activities. They also developed, in collaboration with CDC, summary tables listing all annotated LOIs. The Tanzania team worked closely with CDC team members on qualitative data analysis, use of geographic information systems, and cartography to learn new skills in analyzing the qualitative data and geocoding the LOIs. These dedicated data management and analysis efforts led to detailed, formal spatial datasets of the LOIs and routes and initial qualitative thematic analyses. Although a compiled report from each event or group of events took longer to create, the team could use those results to develop improved visualizations for already-defined and future program goals.

The Uganda team focused on developing detailed FGD and KII transcripts after each event, along with detailed summary tables of all mentioned and annotated LOIs and travel routes. This process was supported by dedicated team members who had expertise in qualitative and spatial data management. The team analyzed the qualitative data for themes about reasons and routes for movement. The spatial analysts and cartographers geocoded the results, building robust spatial datasets of >1,000 unique LOIs and routes. The team also combined the results with other data-gathering initiatives completed by partners on the team, including Infectious Diseases Institute and Baylor Uganda, to characterize cross-border healthcare-seeking behaviors.

The data management approaches of Tanzania and Uganda led to the ability to develop more broadly effective reports and presentations. They were able to include visualizations to address various MOH objectives identified during the preparation phase or newly identified during pandemic response initiatives.

### Application Phase

All teams used PopCAB results to adapt national, district, and POE-level policies, programs, and resource allocation plans (Table 5). All teams enacted these adjustments using qualitative and spatial information mentioned during the PopCAB events. Despite the different strategies implemented in the visualization phase, all teams continued to use the results from completed events throughout the pandemic. The Tanzania and Uganda teams completed multiple analyses to respond to established and newly identified goals throughout the response, stemming from the approaches they followed to develop robust datasets with the gathered data.

**Table 5:** Summary of the public health and border health strategies for COVID-19 response adjusted by applying PopCAB results*

Topic	DRC	Tanzania	Uganda
Identify locations or hours of operations for new POE or community-based mobile surveillance sites	X		X
Prioritize locations, e.g., health facilities or villages, for enhanced surveillance and associated staff trainings and resource allocation	X	X	X
Incorporate additional sectors, e.g., market vendors, in COVID-19 outreach and mitigation measures	X	X	X
Modify risk communication strategies by incorporating more contextually-relevant information and locations with cross-border		X	X
Adjust the national response plan to include cross-border population movement considerations	X		X
Tailor border health system lockdown measures			X
Provide provincial and district surveillance and border health officers with data about movement patterns to tailor surveillance	X	X	X
Prioritize cross-border surveillance committees for enhanced action	X	X	X

*PopCAB was performed May 2020–March 2022. POE, point of entry; PopCAB, Population Connectivity Across Borders.

The teams implemented various contextually-specific initiatives using the results to address consistent and unique objectives. The DRC MOH used PopCAB results from FGDs in and around Kinshasa to identify 3 urban locations for new mobile COVID-19 surveillance to increase the ability to detect illness at key traveler congregation points. In addition, the DRC MOH maintained a plan to routinely conduct PopCAB in Kinshasa to guide when and where to adjust the locations of those mobile urban surveillance sites. They also rapidly applied results to extend operating hours for certain POEs to accommodate unique movement patterns identified through the FGDs.

The Tanzania Port Health unit identified areas for increased engagement with village committees and security authorities to strengthen border health surveillance. As part of that effort, they identified community health workers in areas with increased cross-border connectivity in Dar es Salaam and Tanga Provinces and provided them with additional training on event-based surveillance. The MOH also used the data to select high-traffic locations where they enhanced community outreach and installed handwashing stations.

District-level officials in Uganda on the Tanzania border worked with owners of nighttime bars visited by persons from across the border to increase COVID-19 surveillance. The Uganda team also identified mobile phone market vendors that serve cross-border communities to support them in distributing COVID-19 risk communication materials to high-priority population groups. Along that border and the western border with DRC, the Uganda team applied results to identify schools and markets preferred by cross-border communities for enhanced risk communication in preparation for and during the COVID-19 response lockdown. Like Tanzania, Uganda also applied results to identify village health volunteers who worked in areas with increased cross-border connectivity, including those along routes used by persons fleeing conflict from DRC into Uganda, for prioritized refresher training on community-based surveillance.

All of the teams applied the results from the binational PopCAB events during cross-border meetings to prioritize official and unofficial POEs and other LOIs along travel routes across their shared borders for enhanced risk communication and traveler surveillance. They also prioritized groups of contiguous administrative areas in cross-border surveillance zones for more robust and sustained collaboration and information sharing.

## Discussion

Throughout the COVID-19 pandemic, the DRC, Tanzania, and Uganda MOHs, in collaboration with CDC and other partners, adapted bilateral, national, and local-level strategies to their complex, cross-border sociocultural contexts by gathering and analyzing community-level information on population movement patterns. Although the countries’ MOHs developed implementation goals and plans independently, all 3 followed consistent approaches for developing multisectoral collaboration for participation and applying the results. However, they differed in the intensity of data management and analysis methods, reflecting varying availability of resources including staff time and expertise. Those unique analytic approaches influenced the magnitude of tabulations of locations and routes and qualitative analytical results compiled in formal datasets. Regardless of the depth of analyses, the countries, each with unique COVID-19 epidemiology, border health and public health policies and infrastructure, and cross-border dynamics, provide various examples of ways to incorporate population mobility, a key driver of communicable disease spread, into mitigation measures. Despite differences in data compilation and analysis, their approaches highlight opportunities to achieve impacts across varying staff and financial commitments for creating qualitative and spatial databases.

The teams’ experiences revealed challenges with implementing PopCAB overall and during a pandemic. Although the resources needed to implement any one PopCAB event is low, requiring only field travel support and person-time from the implementers and participants to complete the 1.5-hour event in addition to a printed map and supplies such as markers, pens, and paper, leadership had to secure additional funding and person-time to train staff on the toolkit and to process the gathered data. In addition, the teams had to adhere to COVID-19 mitigation measures preventing in-person trainings and field events at different times throughout the pandemic. To address these considerations, the teams designed and employed online training techniques. They also ensured that previously-trained staff participated in subsequent PopCAB implementation over the 2 years with minimal refresher training, introducing a few new staff at a time rather than entirely new field teams. The teams also adjusted field-based protocols to incorporate COVID-19 mitigation measures during FGDs and KIIs, including physical distancing, with only the facilitator annotating the maps, and use of cloth face coverings. Also, highlighted by the DRC MOH’s approach, teams adjusted expectations for data processing and analyses to accommodate the available resources.

The teams were not able to attribute trends in COVID-19 epidemiology in their countries to changes they made to mitigation measures and policies using PopCAB data because of the complexity of SARS-CoV-2 translocation among mobile populations and difficulty differentiating travel-associated spread from domestic transmission. However, national MOH leadership overseeing these PopCAB activities expressed confidence that their interventions more appropriately supported their communities because they were adapted to the unique, multisectoral and sociocultural environments and were designed through community-engagement efforts. In addition, district-level leadership participated in the initiatives ensuring continuity of these efforts in local programming and resource allocation decisions.

The 3 governments in East and Central Africa implemented community-engagement efforts using the PopCAB toolkit following various staff-time and data management approaches over 2 years of the pandemic to design COVID-19 mitigation strategies. More specifically, they adjusted public and border health policies and programming to address formal and informal cross-border movement patterns, to enhance surveillance and capacity building at newly identified community-based locations and healthcare facilities, and to strengthen cross-border collaboration between neighboring countries. Those MOH-led, community-based initiatives can complement other analytic methods using existing travel and mobility data to incorporate community dynamics more accurately in border health and other preparedness and response strategies for COVID-19 and other communicable diseases. Furthermore, the MOHs can continue to apply the results to other public health goals, including broader outbreak preparedness strategies and cross-border collaboration priorities. Their experiences reveal options government leadership can follow to integrate population mobility and sociocultural factors into public health preparedness and response strategies.
